# Impact of home literacy environment on literacy development of children with hearing loss: A mediation model

**DOI:** 10.3389/fpsyg.2022.895342

**Published:** 2022-10-14

**Authors:** Qianqian Wang, Minjie Ma, Yan Huang, Xichen Wang, Tingzhao Wang

**Affiliations:** Department of Special Education, Faculty of Education, Shaanxi Normal University, Xi’an, China

**Keywords:** children with hearing loss, home literacy environment, literacy development, reading interest, parent-child relationship

## Abstract

Reading presents an unsolved difficulty for children with hearing loss and research on factors influencing their literacy development is very limited. This work aimed to study the influence of home literacy environment (HLE) on literacy development of children with hearing loss and explore possible mediating effects of reading interest and parent-child relationship. 112 Chinese children with hearing loss were surveyed for scales of HLE, literacy development, reading interest, and parent-child relationship. Result analysis showed that HLE significantly predicted literacy development of children with hearing loss and this effect was no longer significant after including reading interest and parent-child relationship as variables. Further, HLE significantly predicted reading interest and parent-child relationship, each of which predicted literacy development and played a significant mediating role in HLE’s influence on literacy development. These findings provide educational tips for families of children with hearing loss.

## Introduction

Reading is an important field of children’s development. Children’s successful reading has a positive and important impact on their growth and development (Baroody and Diamond, 2012). However, there are many factors affecting children’s reading development, including social, cultural, genetic and other aspects (Parrila and Mcquarrie, 2015). Among them, the family factor is one of the most important ones. Taylor and his colleagues have put forward an academic socialization theory and its conceptual framework after an in-depth review of family factors by integrating previous studies on children’s academic and literacy development under the family background (Taylor et al., 2004). This theory proposed that home literacy environment (HLE), and parental involvement and cognition influence children’s reading achievement and the influencing process is complex and diverse, so it is worth exploring the intermediary factors in this influence relationship (Zadeh et al., 2010).

Children with hearing loss represent a special group of children who are unable to hear and cannot normally communicate with the outside world due to physical damage of their hearing system. In spite of continuous development of cochlear implant and other technologies, which help children with hearing loss to get sound (Qi and Mitchell, 2011), there is still a big gap in reading and writing between this group of children and children with typical levels of hearing (Reynolds and Werfel, 2020). Children with hearing loss receive information in a variety of ways, including oral, sign and written language. Studies on children with hearing loss in alphabetic language environments have shown that through the combination of oral and sign language, the understanding of young children with hearing loss can reach the level of children with typical hearing (Mastrantuono et al., 2017). However, there are obvious differences between English and Chinese orthographies. Whereas English uses an alphabetic script in which letters correspond to phonemes, Chinese uses an ideographic script in which the main graphic unit, the character, corresponds to a syllabic morpheme (Shu, 2003). Studies in the Chinese context have shown that children with hearing loss have limited understanding in oral and sign language, while they can use written language to better communicate with people with typical hearing (Feng and Fanlin, 2021). At present, reading is still an unsolved difficulty for children with hearing loss. Further, some studies have shown that early literacy activities can provide important help for literacy development of children with hearing loss (Martindale, 2007). Thus, this study aimed to explore the factors that affect literacy development of children with hearing loss from the perspective of home environment in the Chinese context.

### Home literacy environment

Reading plays a positive role in children’s development. Researchers have been exploring the factors that influence children’s literacy development in order to develop strategies to promote children’s literacy development (Korat et al., 2013). Among many factors that influence children’s literacy development, HLE is one of the most studied (Umek et al., 2005; Niklas and Schneider, 2013). HLE generally refers to the material resources provided by the family for children and the family activities to stimulate children’s language development, such as family books, literacy stimulating activities between parents and children, children’s opportunities to visit libraries, etc. (Umek et al., 2005; Niklas and Schneider, 2013; Boerma et al., 2018). Many studies on children with typical levels of hearing have shown that HLE has a positive impact on their literacy development in alphabetic language context (Carretti et al., 2009; Boerma et al., 2018; Guzmán-Simón et al., 2020). Similar conclusions have been drawn on children with typical levels of hearing in the Chinese context (Li and Tan, 2016; Ho and Lau, 2018).

Researchers have also begun to explore the impact of HLE on literacy development of children with hearing loss (Twitchell et al., 2015; Andrews et al., 2017; Reynolds and Werfel, 2020). Socioeconomic status (SES) is closely related to HLE. Early studies on SES’s influence on literacy ability of children with hearing loss have shown that SES is an important predictor of their reading ability development (Twitchell et al., 2015). A study on HLE and literacy development of 22 children with hearing loss and 27 children with typical levels of hearing has shown that family reading investment on children with hearing loss, especially in books, is significantly related to children’s literacy ability (Reynolds and Werfel, 2020). Further, an intervention activity that provided children with hearing loss with a 1-year reading intervention, including story reading and shared reading activities 20 times a week, has shown that the increase of reading participation has a significant impact on the improvement of children’s literacy skills (Andrews et al., 2017). Although these studies have suggested that HLE has a positive impact on literacy development of children with hearing loss, none has explored how it influences the latter. This study aimed to further explore not only their relationship, but also factors to mediate this process.

### Reading interest

Reading interest is a positive or negative reaction to reading activities (Dobbs-Oates et al., 2015; Boerma et al., 2018). Studies on children with typical levels of hearing in English context have shown that children grown up in rich HLE have better language and literacy ability in the future if they read more often (Baroody and Diamond, 2012; Yeo et al., 2014; Carroll et al., 2019). Similarly, in the Chinese context, the study on family and school related factors influencing their literacy development have shown that reading interest has the most significant predictive effect on children’s reading performance, accounting for approximately 11–21% of variance (Ho and Lau, 2018).

Currently, there are only a few reports devoted to reading interest of children with hearing loss. The study on literacy interest and reading orientation of preschoolers with hearing loss in parent-child reading activities has shown that these children should be given more opportunities to choose the reading content before reading, and they are more interested in manipulative books than in narrative books (Kaderavek and Pakulski, 2007). Similar conclusions have been drawn with another study demonstrating that games matching with the theme of storybooks can effectively improve children’s interest to participate to read (Pataki et al., 2014). These findings remind us that parents’ varied reading activities can promote reading interest of children with hearing loss. However, these results do not directly indicate the specific role of reading interest in children’s reading process. Therefore, the role of reading interest in influencing HLE’s impact on literacy development needs to be further explored.

### Parent-child relationship

Parent-child relationship is the relationship between parents and children. As a key factor in HLE, it is the basis of children’s positive development and healthy growth (Oden et al., 1993). Research on home reading of children with typical levels of hearing has shown that good parent-child relationship can positively promote almost all aspects of children’s reading development (Bus et al., 1995; Bingham, 2007; Hutton et al., 2017). A recent study in the Chinese context has shown that parents’ education levels are positively correlated with the frequency of parent-child literacy activities and children’s views on the quality of parent-child relationship, while the latter two are significantly and positively correlated with each other (Li et al., 2020). Currently, the influence of parent-child relationship of children with hearing loss on their literacy development remains unexplored. However, there are several reports on related topics. A study analyzing the reading and writing characteristics of 12 preschool children with hearing loss during mother-child storybook interaction showed that children with hearing loss are very interested in changing activities in the process of parent-child shared reading (Kaderavek and Pakulski, 2007). In another study, 12 videos of shared reading activities of children with hearing loss with their parents showed that children with hearing loss can quickly adapt to and accept this form of reading activity (Watson and Swanwick, 2008). Conversely, a report by Reynolds and Werfel (2020) showed that parents’ effort to promote the literacy of preschool children with hearing loss has little impact on children’s reading performance. Thus, the role and significance of parent-child relationship in influencing the impact of HLE of children with hearing loss on their literacy development need to be further explored.

**FIGURE 1:**
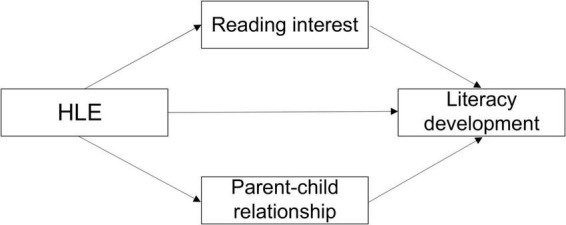
A hypothetical model of children with hearing loss for mediating the interactions between HLE and literacy development by reading interest and parent-child relationship.

As described above, it is suggested that HLE, reading interest and parent-child relationship are important factors to influence literacy development of children with hearing loss, but how they work during this process is not very clear. As we know, this group of children mostly are slow learners and we cannot assume the way for these factors to influence literacy development is the same as that for children with typical levels of hearing. In this study, we hypothesize that HLE of children with hearing loss may positively affect their literacy development, and that reading interest and parent-child relationship play in parallel mediating roles in the regression relationship between HLE and literacy development. The hypothetical model is shown in Figure 1.

## Materials and methods

### Procedures

Approval to our study involving human participants was obtained from the Institutional Research Board of Shaanxi Normal University. Our research team contacted deaf schools from four different regions in China and initiated a research recruitment initiative with the help of school teachers to solicit participants for this study. It is generally believed that HLE has a greater influence on the reading development of pre-school and early stage of elementary children (Baroody and Diamond, 2012; Carroll et al., 2019). In special schools for deaf students in China, children’s grades are divided by considering several factors that include language communication ability and intellectual development. Therefore, our study included children from preschool to grade 3 as our participants, ranging in age from 2 to 13 years old (see “Participants” section for detail). After obtaining the consent of the participants’ parents, the families meeting the conditions of this study (children should be from preschool to grade 3) were finally recruited as the subjects. With the help of teachers from these deaf schools, the survey questionnaires were distributed to the families who agreed to carry out this research. The questionnaires that included the questions on HLE, literacy development, reading interest, parent-child relationship (see below), and other relevant background information such as household income, parents’ education, etc. were completed by parents of the children with hearing loss. In total, 118 questionnaires were obtained, of which 6 did not meet the requirements of the participants (in addition to hearing loss, these children had other serious obstacles and could not carry out reading activities). Therefore, 112 valid questionnaires were finally obtained.

### Participants

The finally recruited 112 children with hearing loss in this study were all from China’s special education schools for the deaf from Heilongjiang (13), Shandong (45), Shaanxi (35) and Xinjiang (19) provinces. They were aged from 2 to 13 years old, including 41 (36.61%) from 2 to 6 years old, 38 (33.91%) from 7 to 9 years old and 33 (29.48%) from 10 to 13 years old. The mother tongue of all these children was Mandarin and they lived in areas inhabited by Mandarin-speaking population. The SES of the subjects’ families were classified according to relevant studies (Liu et al., 2020), as shown in Table 1. The gender distribution was relatively uniform, with 54 boys (48.2%) and 58 girls (51.8%). The grade distribution was from preschool to grade 3, with 52 (46.4%) from preschool (2∼8 years old), 15 (13.4%) from grade 1 (7∼10 years old), 21 (18.8%) from grade 2 (8∼13 years old) and 24 (21.4%) from grade 3 (8∼13 years old). Children with hearing loss attending schools for the deaf are generally older than typically-developing children due to their limited language skills caused by impaired hearing (see below).

**TABLE 1:** Subjects’ family SES characteristics.

	% (of total)
**Monthly household income**	
Low income families	51.79
Medium income families	21.43
High income families	26.78
**Mother’s education level**	
Graduates from senior high schools or technical secondary schools or below	76.79
Graduates from junior colleges (night and TV universities)	13.39
College graduates	7.14
Postgraduates (master or doctor)	2.68
**Father’s education level**	
Graduates from senior high schools or technical secondary schools or below	78.57
Graduates from junior colleges (night and TV universities)	13.39
College graduates	5.36
Postgraduates (master or doctor)	2.68

Monthly household incomes for low, medium and high income families were <4,000, 4,000–8,000, and >8,000 yuan, respectively.

All the subjects had severe hearing system and function loss, being diagnosed with level I and II hearing disability and they all had disability certificates for hearing loss approved by the state. According to the Chinese criteria for hearing disability assessment^1^, hearing disability refers to the hearing loss of both ears to varying degrees due to various reasons, and the inability to hear or hear clearly the ambient sound and speech sound (those who fail to recover after treatment for more than 1 year). Level 1 refers to the average hearing loss of children’s better ears being >90 dBspL with the speech identification rate of <15%, while level 2 defines the average hearing loss of children’s better ears as being 71–90 dBspL with the speech identification rate of 15–30%. It should be noted that none of the children with hearing loss participated in this study was intellectually impaired. They all had been trained in the rehabilitation services and they normally wore hearing aids, or cochlear implants for communication. The language skills of these students were similar for the same grade. Nevertheless, children with either level of hearing disability were severely limited in understanding and communication. Among them, the preschool children with hearing loss could not use sign language at all, while school-age children could barely use sign language. All the subjects used spoken language and supplemented with some sign language to communicate with their parents during daily life.

### Measures

#### Home literacy environment scale

The HLE scale developed by Umek et al. (2005) was used in this study. This scale has been used in related studies on Chinese children with typical levels of hearing (Lu et al., 2018) and for children with intellectual disabilities (Wang et al., 2021), showing high reliability and validity. The scale comprised 33 items in total with five components: stimulation to use language and explanation, reading books to the child visiting the library and puppet theater, joint activities and conversation, interactive reading, and zone-of-proximal-development stimulation. Due to the different language expression and the unique Chinese characteristics, four questions were revised as detailed in our recent study (Wang et al., 2021). For example, “I correct my child’s use of dual and plural, and encourage her to use them correctly” in the original scale by Umek et al. (2005) was changed to “I correct the child’s use of polyphonic words in time, and encourage her to use them correctly.” The Likert-5 point method was used to score these questions, with the scores ranging from “1 (never)” to “2 (rarely),” “3 (sometimes),” “4 (often),” and “5 (always).” The total score was the sum of the scores of all items. The higher the total score, the better the HLE. The reliability analysis showed that the internal consistency reliability of the scale and its components was high, with Cronbach α coefficient of 0.98.

#### Literacy development scale

Parents’ evaluation has been used as a valid assessment of the literacy development of disabled children (Lanter et al., 2012; Westerveld and van Bysterveldt, 2017). The literacy development scale used in this study also required parents to make corresponding evaluation on literacy development for their children with hearing loss. The content of this scale was the evaluation of vocabulary and expression of children with special needs. The scale has been used for evaluation of literacy development of children in China, yielding highly reliable results (Wang et al., 2021, 2022). It comprised 5 items, with a single factor structure. The five questions were (i) Home reading experience has enabled your child to understand more words, (ii) Home reading experience has enabled your child to express sentences more clearly, (iii) Home reading experience has enabled your child to know some new nouns, (iv) Home reading experience has improved your child’s common sense of life, and (v) Home reading experience has improved children’s reading ability. The Likert-5 point method was used to score these questions, with the scores ranging from “1 (completely inconsistent)” to “2 (slightly inconsistent),” “3 (partly consistent),” “4 (mostly consistent),” and “5 (completely consistent).” The total score was the sum of the scores of all items. The higher the calculated total score, the higher the literacy development. The reliability analysis showed that the internal consistency reliability of the scale with its components was high, with Cronbach α coefficient of 0.94. The exploratory factor analysis was carried out and the results showed that only one factor was extracted from the questionnaire, the cumulative interpretation rate was 80.64%, and a load of each item was between 0.85 and 0.93, indicating good structural validity of this scale (Chung et al., 2004). Further confirmatory factor analysis supported the single factor structure of the scale (χ^2^/*df* = 1.382, IF = 0.996, TLI = 0.992, CFI = 0.996, RMSEA = 0.059) (Chung et al., 2004).

#### Reading interest scale

The reading interest scale, developed by Yeo et al. (2014) was used in this study. This scale comprised 6 items, with a single factor structure. It has been previously used in related reading studies of Chinese children with typical levels of hearing (Lin, 2018). Here are two examples of these questions: “When being read a book, my child appears to be interested” and “My child looks at books by him/herself.” The Likert-5 point method was used to score these questions, with the scores ranging from “1 (never)” to “2 (rarely),” “3 (sometimes),” “4 (often),” and “5 (always).” The total score was the sum of the scores of all items. The higher the calculated total score, the higher the reading interest. The internal consistency reliability of the scale was high, with Cronbach α coefficient of 0.93.

#### Parent-child relationship scale

The parent-child relationship scale, developed by Buchanan et al. (1991) was used in this study. This scale has been previously used in related reading studies of Chinese children with typical levels of hearing (Zou, 2016). Of note, this scale was originally designed to be completed by children themselves to evaluate the relationship with their parents. In this work, we revised it to the form of expression to be completed by the parents. For example, we changed the original question “How openly do you talk with your [mother/father]” in the original scale by Buchanan et al. (1991) to “How openly does your child talk with you?,” In addition, we did not include the question “How careful do you feel you have to be about what you say to your [mother/father]?” The revised scale comprised 9 items, with a single factor structure. The Likert-5 point method was used to score these questions, with the scores ranging from “1 (completely inconsistent)” to “2 (slightly inconsistent),” “3 (partly consistent),” “4 (mostly consistent)” and “5 (completely consistent).” The total score was the sum of the scores of all items. The higher the calculated total score, the closer the relationship with parents. The internal consistency reliability of the scale was high, with Cronbach α coefficient of 0.93.

### Data analysis

All study analyses were implemented in SPSS version 24. Descriptive statistics (mean and standard deviation) of each variable (HLE, literacy development, reading interest and parent-child relationship, etc.) were obtained by analyzing the total sum of each variable.

To test the correlations between each pair of these variables, the descriptive values of each variable were then analyzed in pairs for significant difference analysis by the variance analysis built in the SPSS package, with *p* < 0.05 defining as significantly difference.

In order to assess the regression relationship between HLE and literacy development, and the mediating effect of reading interest and parent-child relationship as mediators in that regression relationship, we used Hayes (2013) PROCESS macro program built in the SPSS software package^2^ to test them. This program used ordinary least squares estimation to compute model parameters for various path models (e.g., mediation), such as path regression coefficient, which was analyzed for subsequent significant difference by *t*-test, with *p* < 0.05 defining as significantly difference. In brief, the total scores of HLE, literacy development, reading interest and parent-child relationship were first standardized. Children’s age and gender, parents’ education levels, as well as monthly household income were included as control variables, and the total effect path regression coefficient of HLE on literacy development was analyzed for significance. Further, to explore possible mediating roles of reading interest and parent-child relationship, these two variables were then added to the regression equation, and the total effect path regression coefficients of HLE on reading interest and parent-child relationship, as well as that of reading interest and parent-child relationship on literacy development, and that of HLE on literacy development (in the presence of the added two variables) were analyzed for significance.

To examine the reliability of the mediating effects of the testing variables, the bias-corrected bootstrap confidence interval (CI) built in the SPSS package was calculated with 5,000 bootstrapping samples. The level of the CIs was set at 95% and that of the tests at 5%.

## Results

### Descriptive statistics of home literacy environment, reading interest, parent-child relationship and literacy development

The mean, standard deviation, and difference correlation coefficient of HLE, reading interest, parent-child relationship, and literacy development of children with hearing loss were statistically performed for correlation analysis. Table 2 shows that there was a significant positive correlation between each pair of HLE, reading interest, parent-child relationship and literacy development, which supports the subsequent hypothesis test.

**TABLE 2:** Correlation analysis results of variables.

Variable	*M*	*SD*	1	2	3	4
1. HLE	121.00	29.19	1			
2. Reading interest	20.06	4.45	0.76*	1		
3. Parent-child relationship	18.71	5.98	0.71*	0.58*	1	
4. Literacy development	18.45	4.76	0.70*	0.74*	0.66*	1

*p < 0.05.

### The mediating role of the reading interest and parent-child relationship

To study the mediating role of reading interest and parent-child relationship in HLE’s influence on literacy development, the total scores of each of the four variables (HLE, literacy development, reading interest and parent-child relationship) were standardized. First of all, by controlling for the children’s age and gender, parents’ education levels, as well as monthly household income, the total effect path regression coefficient of HLE on literacy development was significant (β = 0.72, *t* = 10.58, *p* < 0.05), indicating that HLE significantly predicted literacy development. Second, the two variables, reading interest and parent-child relationship, were added to this regression equation. Regression results in Table 3 show that HLE significantly predicted reading interest (β = 0.78, *t* = 12.25, *p* < 0.05) and the latter significantly predicted literacy development (β = 0.46, *t* = 5.19, *p* <0.05). Table 3 also shows that HLE significantly predicted parent-child relationship (β = 0.71, *t* = 10.21, *p* < 0.05) and the latter significantly predicted literacy development (β = 0.29, *t* = 3.57, *p* < 0.05). However, HLE as an independent variable had no significant effect on literacy development, as it did in the absence of the two additional variables (β = 0.15, *t* = 1.48, *p*> 0.05). The above results indicate that reading interest and parent-child relationship play a complete mediating role in HLE’s influence on literacy development and both play in parallel mediating roles in the regression relationship between HLE and literacy development.

**TABLE 3:** Testing the mediation model of HLE’s influence on literacy development.

Regression equation (*n* = 112)		Fitting index		Significance of regression coefficient	
Result variable	Predictive variable	*R* ^2^	*F*	β	*t*
Reading interest	HLE	0.77	25.64	0.78	12.25*
	Age			–0.02	–0.79
	Monthly household income			0.02	0.43
	Mother’s education level			–0.09	–1.07
	Father’s education level			0.04	0.50
	Gender			0.19	1.52
Parent-child relationship	HLE	0.72	18.89	0.71	10.21*
	Age			0.01	0.42
	Monthly household income			0.02	0.57
	Mother’s education level			–0.07	–0.79
	Father’s education level			0.11	1.20
	Gender			–0.01	–0.05
Literacy development	HLE	0.82	26.28	0.15	1.48
	Reading interest			0.46	5.19*
	Parent-child relationship			0.29	3.57*
	Age			0.04	1.59
	Monthly household income			–0.01	–0.38
	Mother’s education level			–0.11	–1.47
	Father’s education level			0.01	0.04
	Gender			–0.03	–0.28

*p < 0.05.

**FIGURE 2:**
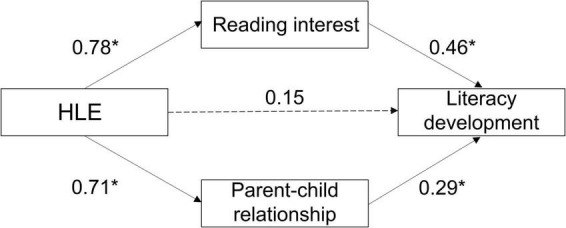
Testing the model of children with hearing loss for the mediating effect of reading interest and parent-child relationship on the interaction between HLE and literacy development. *p < 0.05.

Mediating effect analysis showed that the direct effect of HLE on literacy development was 0.15, and its bootstrap 95% confidence interval included 0, and that reading interest and parent-child relationship each played an intermediary role between HLE and literacy development with a mediation effect value of 0.57. Then, reading interest and parent-child relationship were incorporated into the regression equation to obtain the path model and the results were shown in Figure 2 and Table 4. Analysis of the mediating effect of reading interest between HLE and literacy development showed that the indirect effect of reading interest on HLE’s influence on literacy development was 0.36, and its bootstrap 95% confidence interval did not include 0, indicating that the intermediary effect of reading interest on HLE’s influence on literacy development was significant. The indirect effect of parent-child relationship between HLE and literacy development was 0.21, and its bootstrap 95% confidence interval did not include 0, indicating that the mediating effect of parent-child relationship between HLE and literacy development was also significant. In addition, the difference between the mediating effect of reading interest and parent-child relationship was 0.15, but its bootstrap 95% confidence interval included 0, indicating that the effect of reading interest on HLE’s predictive effect on literacy development was not significantly greater than that of parent-child relationship.

**TABLE 4:** Analysis of the mediation effects of reading interest and parent-child relationship.

Effect type	Effect value	Boot SE	Boot CI lower limit	Boot CI upper limit	Relative effect (%)
Direct effect	0.15	0.10	–0.05	0.36	20.83%
Total indirect effect	0.57	0.11	0.35	0.79	77.78%
Reading interest	0.36	0.09	0.19	0.55	50.00%
Parent-child relationship	0.21	0.06	0.06	0.32	29.17%
Reading interest-parent-child relationship	0.15	0.11	–0.06	0.37	

## Discussion

This study explored the relationship between HLE of children with hearing loss and their literacy development and concluded that there was a significant positive correlation between them. After adding reading interest and parent-child relationship into the regression equation, the prediction of HLE on literacy development decreased significantly. The mediation test showed that there were two independent paths in this mediation process: reading interest and parent-child relationship. These results verified our hypothesis proposed in Figure 1.

This study showed that HLE predicted the literacy development of children with hearing loss. We randomly selected families of young children with hearing loss in four regions of China. Although neither their family income nor their parents’ education levels were high, the HLE average scores were high. This conclusion is consistent with the previous studies on HLE of children with hearing loss in the Chinese and other language environments (Li and Tan, 2016; Ho and Lau, 2018; Guzmán-Simón et al., 2020). Our conclusion that HLE predicted the literacy development of children with hearing loss, confirms and enriches relevant studies of HLE on children’s literacy development (Carretti et al., 2009; Twitchell et al., 2015; Andrews et al., 2017; Boerma et al., 2018), further demonstrates that HLE also positively influences literacy development of children with hearing loss in the Chinese context, and emphasizes the important role of HLE on children with hearing loss in general. However, when two mediating variables were introduced, this regression effect was no longer significant and HLE did not directly affect literacy development of children with hearing loss, further indicating the significance of our study on the mediating role of reading interest and parent-child relationship in this regression relationship. This may be due to the strong dependence of children with hearing loss on their parents, and their parents are generally more protective, compared to the parents of their peers with typical levels of hearing. Moreover, there were more young-age children in the composition of subjects in this study, and reading interest and parent-child relationship may play an important role on their reading choices. Therefore, for younger children with hearing loss, in addition to providing children with rich HLE, their parents should pay more attention to other factors such as reading interest and parent-child relationship that affect the development of children’s reading ability.

The current study demonstrated that parent-child relationship plays a significant mediating role in the relationship between HLE and literacy development of children with hearing loss. This result is essentially consistent with the relevant research results of children with typical levels of hearing (Bus et al., 1995; Bingham, 2007; Hutton et al., 2017; Li et al., 2020), and also consistent with the results of prior related studies on children with hearing loss (Kaderavek and Pakulski, 2007; Watson and Swanwick, 2008). Moreover, our study showed that gender and age cannot predict the regression relationship between HLE and literacy development (see Table 3). The research results from a different language environment suggested a positive role of close interaction between parents and children in children’s literacy development. Of note, the research results of Reynolds and Werfel (2020) showed that investment of literacy materials and shared reading activities of children with hearing loss had an impact on their literacy development, however, parents’ promoting efforts on children’s literacy did not have a significant impact on children’s reading achievement. The parents’ reading investment variable from the study of Reynolds and Werfel (2020) and the parent-child relationship variable from our study both involved participation of parents and their children, but these two studies are different, with the other study focusing on sharing activities and ours focusing on the relationship of parents and their children. Parents’ promotion and efforts in children’s literacy and parent-child relationship are not necessarily positively correlated. The quality of parent-child relationship may be a more important family factor affecting children’s literacy development. This suggests the importance to improve the quality of parent-child relationship while increasing the investment in HLE of children with hearing loss. Furthermore, objective factors such as different language and cultural environments and sample size should also be considered. In summary, our findings suggest that we should improve the quality of parent-child relationship between children with hearing loss and their parents and strengthen the sustainable interaction of parent-child relationship.

This study showed that reading interest plays a significant mediating role in the relationship between HLE and literacy development of children with hearing loss. This conclusion is similar to the effect of reading interest on the reading achievement of children with typical levels of hearing shown in the previous study in the Chinese context (Ho and Lau, 2018). Our results emphasizing the effect of reading interest of children with hearing loss on their literacy development in home reading supplement the previous studies on reading interest of children with hearing loss in the English context (Kaderavek and Pakulski, 2007; Parrila and Mcquarrie, 2015). Although the difference testing result comparing the effect of reading interest with that of parent-child relationship has not passed the Bootstrap test which indicates the effects of both are not significantly different from each other, the predictive function of reading interest on literacy development of children with hearing loss is still worthy of attention. One of the reasons is that children with hearing loss have much less contacts with the outside world than children with typical levels of hearing, and reading is one of the most important media for them to obtain information and exchange with the outside world (Andrews et al., 2017). When they begin to enjoy reading, they likely hope to get the pleasure of communication this way. In addition, we noted that when the two mediating variables of reading interest and parent-child relationship were introduced at the same time, the effect of the control variable, grade, was also significant (see Table 3), indicating that with the growth of age, the role of reading interest and parent-child relationship in the relationship between HLE and literacy development of children with hearing loss becomes more and more significant. The conclusion is that stimulating reading interest of children with hearing loss and improving their parent-child reading fun are important factors to promote their literacy development.

Nevertheless, our study has limitations. First, for young-age children with hearing loss, evaluation of literacy development is difficult. Therefore, we chose to use parental evaluation, but this evaluation method of reading for children with hearing loss does not reflect children’s literacy development comprehensively and needs to improve in the future. Second, due to the limitations of objective conditions, longitudinal data were not collected for the current study, so the causal relationship between variables should be interpreted cautiously. Therefore, future studies should use longitudinal data to examine and replicate our findings. Third, whether there is an intermediary or regulatory mechanism between reading interest and parent-child relationship in the influencing mechanism of HLE on literacy development of children with hearing loss needs to be further explored in future studies.

## Data availability statement

The raw data supporting the conclusions of this article will be made available by the authors, without undue reservation.

## Ethics statement

The studies involving human participants were reviewed and approved by Institutional Research Board of Shaanxi Normal University. Written informed consent from the participants’ legal guardian/next of kin was not required to participate in this study in accordance with the national legislation and the institutional requirements.

## Author contributions

QW and TW contributed to the conception and design of the study. QW performed the data collection, developed evaluation tools, and wrote the manuscript. QW and MM performed data analysis. TW provided advice with draft improvement. YH and XW assisted with data collection. All authors contributed to the manuscript proofread and approved the submitted version.

## Footnotes

1
http://www.gov.cn/ztzl/gacjr/content_459939.htm

2
http://www.afhayes.com
